# EXPERIENCES OF SENIOR SCHOLARS AND PROFESSORS EMERITI AT THE UNIVERSITY OF MANITOBA: ONLINE SURVEY FINDINGS

**DOI:** 10.1093/geroni/igae098.0988

**Published:** 2024-12-31

**Authors:** Michelle Porter, William Kops, Cornelia Kauenhowen

**Affiliations:** University of Manitoba, Winnipeg, Manitoba, Canada; University of Manitoba, Winnipeg, Manitoba, Canada; University of Manitoba, Winnipeg, Manitoba, Canada

## Abstract

Universities are unique in providing specific positions for retired academics to continue to work without pay. At the University of Manitoba (UM) in Winnipeg, Canada, there are two types of retiree positions: Senior Scholar (term) or Professor Emerita/Emeritus (lifetime/honorary). These positions allow retirees to engage in activities like supervising graduate students and conducting research. Supports (e.g., office or lab space) may be provided. However, anecdotal information and faculty consultations have suggested that new policies/procedures and initiatives may be needed to enhance their experiences and overall benefits for the university. An exploratory, online survey designed by age-friendly university committee members was completed by 78 current and past Senior Scholars and Professors Emeriti. Results revealed the many activities engaged in (most commonly reported: 78.7% writing, 70.7% research) as well as challenges experienced (e.g., parking, transitions resulting in temporary losses of email or other computer-related resources). While most (83.8%) indicated that they felt connected to the University, others indicated that they felt cut-off. Some also expressed the sentiment that “…the university/faculty does not initiate action to take advantage of my wisdom, experience and talents.” Respondents further indicated their acceptance of several possible recommendations for improvements for individuals in their positions, with the most popular being a website to highlight their work and accomplishments. Given the many benefits that they see for themselves and the University based, on their continued efforts in their retiree roles, changes are imperative to improve the age inclusivity for individuals at this career stage at UM.

